# Science in Dentistry

**Published:** 1894-11

**Authors:** 


ARTICLE III.

### SCIENCE IN DENTISTRY.

In the April issue of the *Dental Cosmos* of the present year there appeared a thoughtful editorial on "The Scientific Status of the Dental Profession," in which it was held in effect that in this country, the art of dentistry had outgrown the science; that our profession was tending toward a mechanical rather than a scientific excellence. It is not my intention here to combat or controvert in any sense the opinion of the writer, in which, indeed, I most fully share, but to call attention to certain aspects of the question that appear to me to have been left partly undeveloped, or, at most merely suggested or touched upon.

Dentistry in America has, so far, been a natural growth; it has developed from the needs of our people. The white race, not yet perhaps thoroughly acclimated to our extremes of climate, coming here as pioneers and undergoing the physical changes necessitated by the altered environment— the effects of mixture of races, new habits of diet, and especially a most rapid, and, as it were, abnormal mental stimulus—under all these new conditions, has, with the need, developed the remedy to an extent perhaps greater than in any other part of the civilized world.

The art of dentistry owes more to America than to any other country, as the standing and success of American dentists abroad during the past forty or more years sufficiently demonstrates. It is in this country also that the first systematic efforts at special dental education were made; where the beginning of the elevation of dentistry from an art to a learned profesion was first attempted; and, whatever may be the status of dentistry here or elsewhere, so much must be rightly attributed to the credit of American dentists. The amount of human suffering that has been made unnecessary by the inventive genius of the dental profession of this country is incalculable, even when not taking into account its share in the giving of that priceless boon of surgical anæsthetics to the world.

In the natural evolution of things, however, a new state of affairs has been produced, one that requires a certain change of face on the part of the dental profession, if it is to hold what ought to be its proper place in the scientific world, and in the estimation of the public. while dentistry was only an art we easily held the lead, and it was an unnatural presumption for us to think that progress on the same general lines that had so far lead to success would fail us no more in the future than in the past. We had created practical dentistry as an independent profession in which our pre-eminence was recognized throughout the world.

In doing this however, we have narrowed our field and separated ourselves from those who should recognize us as co-workers in a common field of usefulness. Dental surgery is a branch of medical science; it is really a specialty in the broader field of medicine. While this is the truth which no one who considers it can gainsay, it is practically ignored by the public and by ourselves. There is no reason why a surgeon who limits his practice to the oral region should be less of a physician than one who confines himself to the eye, the nose or throat, or the pelvic organs; the collateral relations of the one are not less extensive than those of the other. Yet, at the present time, a dentist ranks lower in popular estimation, I think we must all admit, than a physician or surgeon.

If I interpret correctly a recent decision, this view has received judicial sanction, and is part of the judge-made law of the land. To-day the dentist stands, to the public, somewhere between the physician and chiropodist; his social position approaches the former; his professional rank as a specialist, outside of the lines of legitimate medicine, is nearer than we could wish to the latter.

I state these facts, as to the unsatisfactory status of profession in certain respects, as a preliminary to what I wish to say as to its present needs, which have been so ably commented upon by the editor of the *Dental Cosmos.* The time has come, it seems to me, for us to take a higher stand to elevate our specialty, not only in popular reputation but in fact we have one great advantage: the prestige of American dentistry from its past in good, and is only now threatened by the danger that we underestimate the importance of further progress. The world recognizes our manual skill and invention, and it is not a small matter that the leading teacher of dentistry in Germany, if not on the continent of Europe, should be an American, with all the honors that it is possible for a German university to bestow upon him. We have in this the advantage over our brethren in general medicine, for, notwithstanding what the world owes to American physicians in the progress of medical science, European writers have not yet learned to look to this country leading in the scientific branches of the profession.

That this will be less the case in the future no one who observes the tendencies of American medicine at the present will doubt; and it should be our wish and our earnest effort that American dentistry should also continue the progress it has made. At the present, as Dr. Kirk says, the tendency is too much the other way; "no one who even superficially observes the matter can fail to see that our trans Atlantic *confreres* produce an aggregate of scientific work in dentistry which far exceeds the output in this country in the same lines." This being so, and continuing to be so, it is inevitable that American dentistry must fall in the estimation of scientific men, and, as their dictates are followed invaribly by the reading and thinking public, it must therefore decline still more in popular estimation.

There is no good reason why this should be so, and such reason as exists is not any defence for the actual conditions. Americans are not intellectually behind their co-workers in other lands; the ability to do scientific work is not lacking, nor are there in dentistry the deficiencies that exist in some other departments as compared to those abroad. Our clinical facilities are as extensive as those abroad, and our powers of observation certainly are not inferior. The real difficulty is the lack hitherto of the scientific spirit, and of what I may call the scientific atmosphere, which is the encourager and breeder of the spirit of scientific research. Nevertheless this is coming to us, if it is not here; and, as Dr. Kirk says, there is, even now, "a proportion, small though it may be, of workers in the dental ranks who find or make the time to investigate problems in dental science which have a wider scope and broader application than the direct utilitarian." Were there more of these, and could they impress upon the whole of our profession a little of their spirit, the conditions of which I

now complain could not exist. The trouble is that we have too much adopted what Mr. Howells says is the ideal of our country—business success—as our aim and not kept sufficiently in mind that "wisdom is the principal thing," and that with all our gettitng we should get understanding. We do not seem to understand what scientific work is, or how to go about getting it.

If we wish our profession to stand high in this country we should follow the lead of the regular medical profession that is now in almost every section raising its qualifications, and that has always had an ethical ideal which, although sneered at by the laity, has kept it, even at its lowest stage, within the traditions of a learned professsion. At the present, in all our great centres, like New York, Philadelphia, Boston, Baltimore, etc., there is a large body of physicians acknowledged to be equals of any in the world, and their influence and example are elevating all the rest. I think I am safe in saying that while there yet remains much to be desired the time is coming and will probably be within the lifetime of some of us when there will be centres of medical education in this country that will turn the tide to some extent, and students will cross the Atlantic to sit at the feet of the masters on this side.

The reproach that American medical science is in its "kindergarten" will not be I think much longer justified.

What is needed now is that American dentistry should raise its standard and make its reputation as a scientific specialty of medicine, not a manual art, Hitherto it has been one of the easiest ways to get a living—no thorough educational qualifications, no strict ethical observance, and, in short, no high grade of professional honor or feeling being universally exacted of its followers. Our cities are full of purely commercial dentists, who organize in associations, incorporate themselves for profit, and advertise without scruple or limit. To the general public we are all on one level, and that is apt to be the level of the lowest in its estimation. This is to a great extent due to the fact that some of the teachers in our colleges are men of mercantile propensities, uneducated, and in some instances not only dishonest but unprofessional. We should expect students graduated from such institutions to develop into a lower order of professional life.

To make dentistry a recognized specialty in medicine we will have to demand a medical education in the broadest sense for our dentists, and I believe that it is in this way that true reform will have to be effected. If the dental profession of some State would work for a law modeled somewhat after some of the medical practice acts that are now going into execution, requiring every one who enters the parctice of dentistry after a certain date to show proofs that he has received a liberal and a medical education, and to stand an examination, not only in dental manipulation, but also in the general principles of medical science, we would elevate our profession at once. It would be but a few years until the dentists of that State were appreciated both at home and abroad; they would take rank among their medical *confreres*, and share the advantage of wider association and a broader field of work and usefulness. With a medical education dentists could with a great advantage do a large share of the facial surgery that now passes into other hands.

With such a change the charge that American dentistry is not scientific could not long be justified. Instead of literature "replete with statements and arguments based upon mere speculation,with no foundation of fact beyond that constructed in the brain of the originator," we would begin to have reports judiciously made, original observations and scholarly criticisms upon those of others. Our people are observing and ingenious, and what they most generally lack is not the power of observation, but the education that will enable them to know what to observe. This can only be obtained by study, and can only become general with a higher grade of mental and scientific culture than we have hitherto deemed an essential for membership in our profession. However observant and ingenious a man may be, unless he knows what others have already done, he must waste his mental energies in vain in uselessly going over their work, and the publication of the results of his labors, however strenuous, instead of ranking him with the discoverers, gives him the credit only of his ignorance. There has been a vast amount of misapplied mental labor in this particular direction. I have in mind four men of ability who have contributed largely to the literature of the profession, in which hardly an original idea has been added to advance the progress of our specialty; a lifetime having been wasted in laborious work which has not borne the fruits desired.

Even when really original observations are made, this defective knowledge stands in the way of due credit being given to their author. The world does not look for figs on what it is inclined to consider as thistles, and valuable facts may be ignored or overlooked because they are published where no one looks for them, or because they are hidden among the mass of comparatively worthless material that emanates from so many writers whose only ability is to rehash old facts or emit baseless speculations or commonplace generalities. To be sure this is also done abroad, but there is less of it and at least a reputation of more scholarship and original work. They are now learning to respect American medical science more than was formerly done, and yet "*Americana sunt, non legunter*" is too often their off-hand disposal of really meritorious concontributions from this side of the ocean. Some eighteen or twenty years ago a New York physician published published a paper, giving out views based on observation that within the past few years have revolutionized opinions in regard to an important class of ailments the world over. A german author recently in alluding to them said, in excuse for the neglect they had met with in this country, that they originated in America, where medical science had been up to the present and still in its "kinderschule." The "certain condescension in foreigners," that Lowell satirzed, is often still too manifest in medical matters, and it should be the wish and endeavor of every true American to do away with any just ground on which it can be based. I say nothing of unjust ones, for I trust we are not a people who will willingly submit to injustice.

If American dentistry wishes to keep the rank it has won in the practical development of the art in its present more scientific phases, it must raise its standards of culture, and require of its practitioners a higher grade of requirements, both general and professional, than has heretofore been its rule. I see no better ideal for it than that of being considered as a specialty in the great field of medicine. To be recognized as such we must widen our studies and be able to view our work in all its relations to the human system; a specialist who is not also a well-educated physician is justly not in good standing in the medical profession. I care not what views are held up by others in regard to the study of our specialty, if any advancement is made, it can only be accomplished (as I have said over and over again) by obtaining a broad, liberal, medical education. We must cultivate a liberal professional, rather than a commercial, spirit in our specialty. We may not be able to realize this ideal at once, but we can work toward it, and that it will be attained, if we desire it, I have no doubt. We cannot look to the medical profession to come to us; the lesser cannot include the greater; but it has abundant to take us in, and there is no doubt of its good will if we only accept the conditions it imposes upon its own members. For the present we might look for a friendly appreciation only; an organic union is only a possibility of the future. But by standing still, or following some of our present tendencies, I fear the difference between us as regards professional standing and public estimation, will widen instead of diminish.—*Pacific Coast Dentist*.

